# Emotional wellbeing in intercity travel: Factors affecting passengers' long-distance travel moods

**DOI:** 10.3389/fpubh.2022.1046922

**Published:** 2022-12-15

**Authors:** Xiaowei Li, Yuting Wang, Junqing Tang, Lanxin Shi, Ting Zhao, Jun Chen

**Affiliations:** ^1^School of Civil Engineering, Xi'an University of Architecture and Technology, Xi'an, China; ^2^Jiangsu Province Collaborative Innovation Center of Modern Urban Traffic Technologies, Southeast University, Nanjing, China; ^3^School of Urban Planning and Design, Shenzhen Graduate School, Peking University, Shenzhen, China; ^4^Key Laboratory of Earth Surface Systems and Human-Earth Relations of Ministry of Natural Resources of China, Shenzhen Graduate School, Shenzhen, China

**Keywords:** travel mood, emotional wellbeing, intercity travel, ordered logit model, SVM model

## Abstract

The travel mood perception can significantly affect passengers' mental health and their overall emotional wellbeing when taking transport services, especially in long-distance intercity travels. To explore the key factors influencing intercity travel moods, a field survey was conducted in Xi'an to collect passengers' individual habits, travel characteristics, moods, and weather conditions. Travel mood was defined using the 5-Likert scale, based on degrees of happiness, panic, anxiety, and tiredness. A support vector machine (SVM) and ordered logit model were used in tandem for determinant identification and exploring their respective influences on travel moods. The results showed that gender, age, occupation, personal monthly income, car ownership, external temperature, precipitation, relative humidity, air quality index, visibility, travel purposes, intercity travel mode, and intercity travel time were all salient influential variables. Specifically, intercity travel mode ranked the first in affecting panic and anxiety (38 and 39% importance, respectively); whereas occupation was the most important factor affecting happiness (23% importance). Moreover, temperature appeared as the most important influencing factor of tiredness (22% importance). These findings help better understand the emotional health of passengers in long-distance travel in China.

## Introduction

To meet the needs of passengers' growing travel demands, the national transportation infrastructure in China has been developing rapidly, encompassing 5,198,100; 230,000; 127,700; and 146,000 km of total highways, air routes, waterways, and railways by December 2020, respectively (including 30,000 km of the high-speed railway—HSR). Concurrently, the annual passenger flow volume reached 4 billion travelers, with passengers' intercity traffic accounting for 71.3% of road transport, 22.8% of railway transport, 4.3% of civil aviation, and 1.6% of water transport (1). Corresponding with the increasing demand for intercity travel, passengers have higher requirements for travel service quality, turning greater focus to their own emotional wellbeing during travel. Accordingly, travel mood is an expression of passenger satisfaction regarding their emotional state, and an improved understanding of passenger moods during trips, as well as the factors affecting emotional perception during intercity long-distance travel, is critical for improving nationwide transportation services in a manner consistent with improved traveler mental health.

Namely, *mood* refers to “the emotional interpretation of perception, information, or knowledge” (2). Indeed, many studies related to travel behavior have highlighted the important role of mood on trips (3), as investigating travel moods is a popular topic in the literature. For example, Bel and Jordan (4) argued that passenger preferences were shifting from a focus on basic functional services, to travel mood and pleasure-seeking moments; whereas Jonas (5) argued that the contemporary enjoyable car experience was related to the travel mood and visual stimuli. Alternatively, Stradling et al. (6) noted that bus ride experiences included a variety of non-instrumental travel mood factors, such as scenery, stress, and entertainment. Further, Rui et al. (7) studied passengers' travel mood responses to bus services, Rajesh and Daruri (8) assessed the effect of social cues on passengers' travel moods using comfort, relaxation, and joy, and Meenar et al. (9) studied the relationship between passengers' travel moods and the transport environment, linking various travel mood experiences related to fear, anger, sadness, joy, and anticipation.

Passengers with different individual attributes would undergo various moods when traveling between cities. Therefore, the individual attributes of passengers may be hypothesized to affect the passengers' subjective feelings during intercity travel. In addition, passengers often choose different modes of transportation at different intercity travel distances. The differentiated service quality provided by different modes of transportation facilities may lead to obvious changes in the travel mood of passengers. Thus, the travel attributes, such as travel mode choice and travel distance, may also influence the travel mood of passengers. What's more, weather conditions can also affect the mood of travelers. It's straightforward to realize that good weather can make people feel better when they travel long distance between cities. From what has been discussed above, it could be inferred intuitively that the travel mood of passengers may be affected by the individual attributes of passengers, travel attributes and weather conditions.

With the excellent prior knowledge explored by many previous studies (4–7, 10–12) on people's emotional perception during traveling, it is relatively clear on this topic in urban transportation field. However, little has been known and explored toward a comprehensive investigation investigating the factors influencing passengers' emotional perception over long-distance intercity travels. Moreover, existing studies have discussed the impacts of individual and travel attributes on corresponding moods; nonetheless, the potential impact of transportation mode choices on travel mood has been largely overlooked, especially with respect to the nationwide integrated comprehensive transportation network, including airplanes, high-speed railways (HSRs), conventional railways, and express busses throughout China. Due to the differential service qualities, travel mode choices may potentially impact passenger mood during intercity travel as well. Although some previous studies have explored the influence of weather on passenger mood during intra-city travel (10, 11, 13), it is not known if these same factors are at play during intercity travel as well. Additionally, the vast majority of the cities used as cases are located in developed countries such as the United States and Europe, while few cases exist in developing countries, including China, which has the longest high-speed railway in the world.

Accordingly, to strengthen and fill the aforementioned links and gaps in present understanding, this research used a random sampling survey method to obtain data on passengers' intercity travel activities from Xi'an and matched it with the weather characteristics at the moment of passenger departure. Both an ordered logit model (OLM) and SVM (14–20) were applied in tandem to explore the critical factors affecting passengers' intercity travel emotional perception, and a confusion matrix was used to assess model accuracies for validating the results and implications. The primary contributions of this study can be summarized as follows:

(1) This study explores the relationship between individual passenger's intercity travel moods, personal characteristics, weather conditions, travel mode choices, and intercity travel time, and helps better understand the complexity of emotional wellbeing, and its various determinants during long-distance human mobility.
(2) The SVM and OLM were used to reveal the effect of passenger's personal characteristics, weather conditions, travel mode choices, and intercity travel time on passenger's intercity travel moods, which effectively help to better understand the importance of these factors and their positive or negative effects.
(3) Taking a representative tourist city in China as the case study, this study provides an interdisciplinary lens for better managing mobility travel and tourism, offering insights into different travel emotional perceptions with respect to travel mode (including HSR), purpose (including tourism or leisure), and socio-economic attributes.

The remainder of this paper is organized as follows: Section Literature review presents a literature review; Section Data and material introduces the sources and methods for data collection; Section Methods discusses the developed models and the corresponding model evaluation methods; Section Results provides the results and the main findings of this study, and Section Discussion offers the conclusions.

## Literature review

To date, previous studies have explored the weather (21), travel time (11), travel cost (22), and modes of transport (23) on passengers' travel emotion. Yazdanpanah and Hosseinlon (24) conducted a passenger questionnaire survey at IKIA and adopted a mixed discrete potential class model to explore the factors affecting intercity travel emotions, revealing that both weather and intercity travel cost had a significant influence. Notably, the results showed that passengers were more likely to have experienced negative emotions when traveling in a poor weather condition. Moreover, Alberto et al. (25) applied a binomial logistic model to study the impacts of extreme intercity travel weather conditions on passenger travel emotions between London and Glasgow, UK, which revealed that traffic interruption was most associated with negative passenger emotions. Wu and Liao (26) explored intercity passenger travel in Beijing from 2014 to 2018, concluding that extreme weather greatly affected passengers' travel emotions and reduced their travel demand.

Furthermore, it is found that intercity travel time can also affect passenger travel emotions. For instance, Masson and Petiot (27) explored the HSR in Southern Europe between Perpignan, France, and Barcelona, and used the New Economic Geography model to explore the impacts of intercity travel distance, time, weather, education level, personal monthly income, gender, and built environment on passenger emotion, revealing that intercity travel time had the most significant impact, where the two were strongly negatively correlated. However, the detailed influential effects of those factors remained underdeveloped. Beam et al. (11) examined southwest Atlanta to explore the impacts of intercity travel time, extreme weather, and travel distance on passenger emotions, concluding that passengers with uncertain travel times experienced psychological pressure, and thus suffered from greater irritability.

Travel costs may also impact intercity passenger emotions as well. Delaplace and Dobruszkes (22) interviewed tourists near the Eiffel Tower, Lyon Central Railway Station, and Notre Dame Cathedral in Paris, using a logit model to analyze the influencing factors of passenger travel emotion. Their results showed that ticket price, convenience, and speed were the strongest determining factors. In addition, Harvey et al. (28) investigated British passengers' travel emotions toward intercity travel, similarly revealing that travel cost, reputation, and comfort had the most significant impacts.

Lastly, the importance of transportation mode choice on intercity passenger emotions has also been explored. For example, St-Louis et al. (29) used commuter travel survey data at McGill University in Montreal and adopted ordinary least squares regression to compare the different travel emotions of passengers across six travel modes (walking, cycling, car, bus, subway, and commuter train). The authors found that individual attributes, transportation modes were all the most significant determinants. Specifically, the results showed that slower modes of transportation (walking and cycling) seemed to generate more positive emotions than others, as pedestrians, train commuters, and cyclists reported significantly higher positive emotions than drivers or subway and bus users. Later, Morris and Guerra (23) investigated the emotions of more than 13,000 interviewees engaged in randomly selected intercity travel activities across the United States, using an ordinary least square method and fixed effects panel regression model to explore the impacts of transportation modes on passenger emotions. The results showed that passengers had a better emotional experience when traveling by car than by trains, as individuals experienced feelings of power, mastery, control, prestige, or self-esteem, which showing an interesting findings for the passengers in intercity travel in the US.

In terms of the approach of modeling and analytics, traditional discrete choice models, including the ordered logit model, are often used to explore the influencing factors of intercity passenger travel emotion (30–32). In recent years, machine learning is increasingly used in traffic behavior research (33–38). One such example, SVM, is often used to solve classification and regression problems (39), due to its strong capability to process data classification. In particular, SVMs are often widely applied for the detection (40) and severity level predictions of traffic accidents (41), as well as travel behavior analyses (43). For example, Wang et al. (44) used SVM to predict the short-term traffic flow of expressways, citing that this technique could overcome problems related to over-fitting of data and local minima solutions, in addition to its superior prediction capability than multi-layer feedforward neural network models. Yao et al. (45) applied an SVM to traffic accident detection on expressways and urban main roads, similarly explaining a higher correlated improved accident detection capability in SVMs than multilayer feedforward and probabilistic neural network models. Li et al. (41) examined data points of motorcycle traffic accidents along rural Texas Highway 88, revealing that the average absolute deviation and mean square prediction error measures verified that the SVM outperformed the traditional negative binomial regression model prediction accuracy on accident severity level. Luo et al. (42) discussed the applicability of SVM for assessing the choice of travel mode based on a 1990 travel survey of San Francisco Bay Area residents, concluding that this method can offer improved predictive ability compared to multi-logit or multi-layer feedforward neural network models under different training sample sets. Yang et al. (46) also compared the prediction accuracy of SVM, nested logit, and multi-layer feedforward neural network models based on a 1-day 2005 travel survey of residents in China, showing that the SVM maintained a faster convergence speed and higher predictive accuracy. Lastly, Allahviranloo and Recker (43) used SVM to explore the data from a 2001 California residents' travel survey regarding their daily activity-travel pattern recognition and found it advantageous and outperforms a multinomial logit model in terms of its predictive accuracy.

From the aforementioned literature (readers can also refer to the summary of the representitative studies from above in Table 1), however, we found an obvious research gap regarding passenger emotions in intercity transportation modes (airplanes, HSR, conventional trains, and express buses), especially for HSR—it is more underdeveloped comparing to other tranditional modes. Moreover, while most scholars have discussed the impacts of individual and travel attributes on travel mood, the potential impacts of intercity transportation mode choice and weather on travel mood, however, have largely been in deficiency. In addition, the vast majority of assessed cities are located in developed countries within the United States and Europe, while few cases exist from developing countries, including China, which maintains the world's longest HSR infrastructure and operation system. Lastly, traditional discrete choice models, such as the OLM, have been widely used; however, the feature importance of significant influential factors cannot be extracted and visualized by suchlike tranditional modeling techniques—a more interpreatable machine-learning-combined approach is needed.

**Table 1:** Previous intercity travel passenger emotion research.

**References**	**Dataset**	**Dependent variable**	**Independent variable**	**Method**	**Results**
Yazdanpanah and Hosseinlou (24)	Survey conducted in January–February, 2015 at Imam Khomeini International Airport (IKIA)	Travel emotion	The five personality factors	Hybrid discrete latent class model	Passengers were more likely to experience negative emotions during bad weather; Conscientious individuals considered travel cost more likely than other attributes
Alberto et al. (25)	Internet-based travel behavior survey with >2,000 respondents in London and Glasgow	Travel emotion; travel behavior	Extreme weather; long-distance travel;	Binomial logit	Respondents were generally cautious about traveling during extreme weather events
Beam et al. (11)	Survey data collected at the six-mile buffer zone of the Atlanta Belt Line Eastside Trail; Three-mile transit access zone around three transit stations in southwest Atlanta	Travel emotion	Travel time; extreme weather; long-distance travel	LTS	Uncertain travel times produced psychological pressure, resulting in agitated moods among passengers
Masson and Petiot (27)	Forthcoming South European HSR lines between Perpignan and Barcelona	Tourism attractiveness	Travel distance, education level; car; income; gender; weather; built and natural environment	New economic geography (NEG) models	Under the same time limit, passengers tended to choose the fastest mode of transportation or travel directly to a nearby location, prioritizing short time journey times, and remaining at the destination for long time
Delaplace and Dobruszkes (22)	The Eiffel Tower and Lyon visitors near the central station and the Notre Dame cathedral in Paris	Travel emotion	Ticket price; convenience degree; rail speeds	Logistic regression model	Higher ticket prices negatively affected travel mood
Harvey et al. (28)	Attitudes and perceptions of long-distance travel in the UK	Long-distance travel emotion	Travel security; prestige of HSR; comfort; negative HSR aspects; travel time	Varimax rotation and alphas	Travel costs, as well as the reputation and comfort of high-speed trains significantly impacted passengers' travel mood
St-Louis et al. (29)	Commuter survey carried out at McGill University in Montreal, Canada	Travel emotion; travel satisfaction.	Trip and travel characteristics; personal characteristics; travel and mode preferences	Ordinary least square regression analysis	Slower modes of travel (walking and cycling) appeared to generate more positive emotions than cars and public transport, with pedestrians, train commuters and cyclists reporting significantly higher positive feelings than drivers or subway and bus users

## Data and material

### Field survey

In this paper, we select the Xi'an city as the study area and the destination of our field survey investigation. Xi'an is the capital city of Shaanxi Province in China, the starting point of the famous Silk Road, the core city in the “Belt and Road” strategy, and an important national center for tourism, education, and industry. Xi'an is one of the top tourist destinations in China, and six sites have been listed on the World Heritage Lis.

The questionnaire consists of three parts: First, the individual's socioeconomic characteristics and weather conditions, including gender, age, occupation, personal monthly income, car ownership, temperature, rainfall, relative humidity, wind speed, air quality index, and visibility, Second, passenger travel characteristics, including travel purpose, intercity modes of transport, and travel time. Third, passenger travel mood was defined by happiness, panic, anxiety, and tiredness (10), and classified according to the Likert scale (e.g., five levels of happiness: very unhappy, unhappy, general, happy, and very happy). The detailed design and marking of variables are shown in Table 2; whereas the definitions of travel mood are shown in Table 3.

**Table 2:** Variable definitions and markings.

**Variables**	**Marking or calculation of variables**
Gender	“Male” = 1, “Female” = 0
Age	“0–29” = 1, “30–59” = 2, “≥60” = 3; Unit: Year
Occupation	“Enterprise personnel” = 1, “Institution personnel” = 2, “Students” = 3, “Farmers” = 4, “Self-employed” = 5, “Others” = 6.
Personal monthly income	“ < 3” = 1, “3–4” = 2, “4–5” = 3, “5–6” = 4, “>6” = 5; Unit: Thousand yuan
Car ownership	“No” = 1, “Yes” = 0
Travel purposes	“Mandatory travel ”(e.g., business, returning from holidays) = 0, “Leisure travel” (e.g., tourism, visiting relatives) = 1
Intercity mode of transportation	“Airplane” = 1, “HSR” = 2, “Bullet train” = 3, “Train” = 4, “Express bus” = 5
Temperature	“>25” = 1, “15–25” = 2, “10–15” = 3, “5–10” = 4, “ < 0” = 5; Unit: °C
Happiness degree	“Very unhappy” = 1, “Unhappy” = 2, “General” = 3, “Happy” = 4, “Very happy” = 5
Panic degree	“Very unpanicked” = 1, “Unpanicked” = 2, “General” = 3, “Panicked” = 4, “Very panicked” = 5
Anxiety	“Very un-anxious” = 1, “Un-anxious” = 2, “General” = 3, “Anxious” = 4, “Very anxious” = 5
Tiredness degree	“Very unfatigued ” = 1, “unfatigued” = 2, “General” = 3, “Tired” = 4, “Very tired” = 5

**Table 3:** Definitions of travel moods.

**Variable degree**	**Observation variables**
Happiness	Indicates reasonable traffic policies during travel, that passengers can easily rest and communicate during travel, and the travel environment is comfortable.
Panic	Represents feelings that under the pressure of large passenger flow (i.e., the carrying capacity of the specific transportation mode is in a supersaturated state), may lead to the occurrence of large-scale disorderly crowding, trampling, and other dangerous situations. If an emergency occurs, the passenger believes that they or the people around them will be in danger, resulting in uncooperative and unreasonable psychological and behavioral responses in the face of real or imagined threats.
Anxiety	Depicts that during the process of intercity travel, trains or planes are delayed due to traffic congestion, or some other intercity travel time delay, disrupting passengers schedule due to the tension.
Tiredness	Represents congestion in the process of taking a vehicle or the travel time is too long causing passengers to feel tired.

### Data acquisition

Field survey data was obtained through questionnaires. Investigators conducted field surveys at Xi'an Xianyang International Airport, Xi'an North Passenger Station, Xi'an Railway Station, and Xi'an Bus Station from April 10 to 17, 2020. A random sampling technique was used in all field surveys to ensure uniform distribution across the survey population at different levels. Investigators first explained the purpose of the survey to respondents, then invited them to participate the questionnaire survey. All respondents were assured that the survey was completely voluntary and their data were recorded anonymously. It took ~2 min to fill out the questionnaire and the investigator can respond immediately to any questions the respondents may have.

A total of 2,400 questionnaires were distributed in the survey and 2028 valid questionnaires were obtained (84.5% effective recovery rate) after excluding 372 invalid questionnaires that contain missing answers or non-compliant answers based on the situation and guidelines. *Baidu Map* was used to measure intercity travel distance and travel time according to the operation schedule, origin, destination, and identification number of the selected mode of transportation. Weather information at the time of passenger departure was also obtained. Wind and air quality indices were based on weather forecasts recordings, while temperature, humidity, rainfall, and visibility data were derived from the National Meteorological Data Sharing Platform (47). Description of the category variables and continuous variables are shown in Tables 4, 5, respectively.

**Table 4:** Description of the category variables.

**Categorical variables**	**Categories**	**Unit**	**Marking**	**Frequency**	**Proportion**
**Dependent variable**					
Happiness degree	Very unhappy	/	1	240	11.83%
	Unhappy	/	2	604	29.78%
	General	/	3	839	41.37%
	Happy	/	4	319	15.73%
	Very happy	/	5	26	1.28%
Panic degree	Very unpanic	/	1	147	7.25%
	Unpanic	/	2	278	23.71%
	General	/	3	500	24.65%
	Panic	/	4	829	40.88%
	Very panic	/	5	274	13.51%
Anxiety	Very unanxious	/	1	137	6.76%
	Unanxious	/	2	305	15.04%
	General	/	3	519	25.59%
	Anxious	/	4	832	41.03%
	Very anxious	/	5	235	11.59%
Fatigue degree	Very indefatigable	/	1	168	8.28%
	Indefatigable	/	2	409	20.17%
	General	/	3	650	32.05%
	Fatigued	/	4	644	31.76%
	Very fatigued	/	5	157	7.74%
**Independent variables**					
Gender	Male	/	1	1,002	49.40%
	Female	/	0	1,026	50.60%
Age	0–30	Year	1	1,112	54.80%
	31–60	Year	2	898	44.30%
	60 and above	Year	3	18	0.89%
Career	Enterprise units	/	1	592	29.20%
	The personnel of institutions	/	2	276	13.60%
	Students	/	3	624	30.80%
	Farmers	/	4	148	7.30%
	Self-employed households	/	5	167	8.23%
	Others	/	6	221	10.90%
Personal monthly income	0–3,000	Yuan	1	741	36.60%
	3,000–4,000	Yuan	2	565	27.90%
	4,000–5,000	Yuan	3	260	12.80%
	5,000–6,000	Yuan	4	207	10.20%
	6,000 and above	Yuan	5	253	12.50%
Car ownerships	No	/	0	977	48.20%
	Yes	/	1	1,051	51.80%
Travel purposes	Mandatory travel	/	0	1,032	50.89%
	Leisure travel	/	1	996	49.11%
Intercity mode of transportation	Airplane	/	1	460	22.68%
	HSR	/	2	648	31.95%
	Bullet train	/	3	126	6.21%
	Train	/	4	496	24.46%
	Express bus	/	5	298	14.69%
Temperature	>25	°C	1	96	4.73%
	15–25	°C	2	1,127	55.60%
	10–15	°C	3	547	27.00%
	5–10	°C	4	211	10.40%
	< 0	°C	5	47	2.32%

**Table 5:** Description of the continuous variables.

**Continuous variables**	**Unit**	**Minimum value**	**Maximum value**	**Mean value**	**Standard deviation**
Relative humidity	%	0	90	40.17	21.976
Rainfall	mm	0	54	0.525	2.346
Windpower	Wind level	0	8	2.313	1.01
Air quality index	μg/sqr meter	5	387	101.324	54.6
Visibility	km	216	8,200	5,528.418	858.161
Intercity travel time	h	0	680	6.169	16.554
Intercity travel cost	Yuan	0	5,400	354.056	364.795
Intercity travel distance	km	0	8,748	894.041	646.304

## Methods

The data mining tool Python *pycaret* was used to perform the SVM model, and STATA (*v*.16.0) was used to carry out the ordered logit regression and confusion matrix calculations.

### Ordered logistic regression

To date, the discrete choice model based on the logit model has been widely used in traffic travel-related research, primarily including the logit, probit, and their many variations. As the dependent variables in this study were ordered categorical variables, an ordered logistic regression was selected. Ordered logistic regression is obtained by defining an unobserved variable ***Z***, which can be used as the basis of ordered data modeling. Here, the mood of intercity travelers is an ordinal variable, in which happiness, panic, anxiety, and tiredness were divided into five levels. Each involved variable could thus be expressed by Equation (1):


(1) $$\text{Z=}\beta \text{X}+\varepsilon_{i}$$

where *X* is the vector of the independent variable determining the discrete ordering i for each observation, β is the vector of the regression coefficient, and ε_*i*_ is the random error term (48).

Accordingly, travelers' moods could be expressed by Equation (2):


(2) $$y=\{\;\begin{array}{c} \;1\;when\;Z\;\leq \mu_{1}\;\left(Particularly\;dissatisfied\right)\;\\ \;2\;when\;\mu_{1}<Z\leq \mu_{2}\;\left(Dissatisfied\right)\;\\ 3\;when\;\mu_{2}<Z\leq \mu_{3}\;\left(Generally\right)\;\\ \;4\;when\;\mu_{3}<when\;\mu_{3}<Z\leq \mu_{4}\;\left(Satisfied\right)\;\\ \;5\;when\;\\ \;5\;when\;Z>\mu_{4}\;\left(Particularly\;satisfied\right)\; \end{array}$$

where μ_*i*_ is the unknown estimated parameter of the traveler's mood, and *y* (i.e., the threshold) corresponds to the integer order.

To estimate regression coefficient **β** and estimated parameters μ_*i*_, the random error term ε_*i*_ was assumed to be an independent and identically distributed logistic distribution. Through this hypothetical result, an ordered logistic model was obtained and the probability that passengers' travel moods belong to any one of the five levels was defined by Equation (3):


(3) $$\begin{array}{l} P_{i}=\Omega \left(\mu_{i}-\beta X\right)-\Omega \left(\mu_{i-1}-\beta X\right) \end{array}$$

where *i* = 1, 2, 3, 4, 5, respectively, and **Ω** is the upper and lower limits of the standard logistic cumulative distribution function, while μ_*i*_ and μ_*i*−1_ rendered the outcomes of *i*. Further parameters could be estimated by maximum likelihood. For a population of *N* observations, the log-likelihood function of the ordered logistic model was defined by Equation (4):


(4) $$\begin{array}{l} LL=\overset{N}{\underset{n-1}{\sum }}\overset{I}{\underset{i-1}{\sum }}\delta_{in}\ln\left[\Omega \left(\mu_{i}-\beta X_{n}\right)-\Omega \left(\mu_{i-1}-\beta X_{n}\right)\right] \end{array}$$

where δ_*in*_ is equal to 1 if the observed discrete outcome point *n* is *i*, otherwise it is zero.

An odds ratio (OR) was used to quantify the impacts of the explanatory variables on the outcome. Specifically, the OR was calculated for variables of interest in the ordered logit regressions, where OR results represent the increase in the odds of the outcome if the value of the variable increased by one unit (49, 50). The OR for the *j*^*th*^ variable *x*_*j*_ can be calculated by Equation (5):


(5) $$OR=\frac{odds(X,x_{j+1})}{odds(X,x_{j})}=\frac{\exp(X\text{β})\times \exp(\beta_{j})}{\exp(X\text{β})}=\exp(\beta_{j})$$

### Support vector machine

SVM is a supervised learning method developed from statistical learning theory used for data analysis and pattern recognition and can be used for data classification and regression (51). The present study used SVM as the regression technology, with the basic principle based on non-linear mapping of the data to high-dimensional feature space, and then constructing a regression estimation function within the feature space, before mapping it back to the original space. The non-linear transformation is carried out by defining an appropriate kernel function (52, 53). In addition, considering that some samples cannot be correctly classified by the separation of hyperplanes, relaxation variables were used to resolve this problem (54). Here, it was assumed that the data set had *N* sample spaces, the training set was *D* = {(*x*_*n*_, *y*_*n*_)|*n* = 1, 2, 3, , *N*}, and the regression function was *y* = *w*^*T*^Φ(*x*)+*b*, where Φ(*x*) is the non-linear mapping function. Accordingly, the original space was mapped to a high-dimensional space, and the optimization of the support vector regression model equation was defined by Equation (6):


$$\begin{array}{l} \min\frac{1}{2}||w|{|}^{2}+Z\overset{N}{\underset{n=1}{\sum }}\left(\xi_{n}+\xi_{n}^{*}\right) \end{array}$$

(6) $$s.t.,\{\begin{array}{c} y_{n}-w^{T}•\Phi (x_{n})-b\leq \varepsilon +\xi_{n}^{*}\\ w^{T}•\Phi \left(x_{n}\right)+b-y_{n}\leq \varepsilon +\xi_{n}\\ \xi_{n}\geq 0,\xi_{n}^{*}\geq 0 \end{array}$$

where *w* and *b* are the weight vector and offset, respectively; *Z* is the penalty parameter; ξ_*n*_ and $\xi_{n}^{*}$ are the relaxation variables, and ε is the loss function parameter. The loss of the model was then calculated only when the absolute value of the difference between the predicted and actual values was greater than ε. Notably, the basic form of the above problem is a constrained convex quadratic program. A Lagrange multiplier buffer was introduced, and the constraints were integrated into the objective function using a Lagrange function to solve the dual problems (Equation 7) (55):


(7) $$\begin{array}{l} \max[-\frac{1}{2}\sum_{n=1}^{N}\sum_{r=1}^{N}\left(\alpha_{n}-\alpha_{n}^{*}\right)\left(\alpha_{r}-\alpha_{r}^{*}\right)\Phi \left(x_{n}\right)•\Phi \left(x_{r}\right)\\ +\sum_{n=1}^{N}\left(\alpha_{n}-\alpha_{r}^{*}\right)y_{k}-\sum_{n=1}^{N}\left(\alpha_{n}+\alpha_{k}^{*}\right)\varepsilon ]\\ s.t.,\sum_{n=1}^{N}\left(\alpha_{n}-\alpha_{n}^{*}\right)=0,0\leq \alpha_{n}\leq Z,0\leq \alpha_{n}^{*}\leq Z \end{array}$$

Here, the kernel function determines the mapping relationship between the training samples from the original space to the high-dimensional feature space. In this paper, the RBF radial basis kernel function was used (56) and can be expressed as Equation (8):


(8) $$\begin{array}{l} f\left(x,y\right)=\exp\left(-\eta |{\left|x-y|\right|}^{2}\right) \end{array}$$

where η is the parameter set of the radial basis kernel function.

SHAP is a novel model interpretation method that utilizes the Shapley value from game theory to combine optimal credit allocation with local explanations (57), and was applied here to measure variable feature importance. It can be used in conjunction with different machine learning models for model interpretation. For a factor subset *S*∈*F* (where *F* stands for the set of all factors), two models are trained to extract the effects of factor *j*. The first model *f*_*S*∪{*j*}_(*X*_*S*∪{*j*}_) is trained with factor *j*, while the second *f*_*S*_(*X*_*S*_) is trained without it. Here, *X*_*S*∪{*j*}_ and *X*_*S*_ are the values of input feature factors. The differences in model output *f*_*S*∪{*j*}_(*X*_*S*∪{*j*}_)−*f*_*S*_(*X*_*S*_) are computed for each possible subset *S*∈*F*\{*j*}, and the Shapley value of a factor *j* is calculated *via* Equation (9) (57, 58):


(9) $$\begin{array}{l} \emptyset_{j}=\underset{S\in F\backslash \left\{j\right\}}{\sum }\frac{|S|!\left(|F|-|S|-1\right)!}{|F|!}\left(f_{S\cup \left\{j\right\}}\left(X_{S\cup \left\{j\right\}}\right)-f_{S}\left(X_{S}\right)\right) \end{array}$$

### Confusion matrix

The collected data were randomly divided into calculation and verification sets according to an 8:2 ratio. Assuming that there were *S* travel modes in a data set, different prediction models based on the data will produce different estimated values. Accordingly, to test the accuracy of the prediction models, the calculation and verification datasets can be used, and the prediction results of each model can be summarized in a confusion matrix (Table 6) (59), where *Recall*_*i*_ is the recall rate, *Precision*_*i*_ is the accuracy rate, *Accuracy* is the correct rate, and *N* is the total number of samples (Equations 10–12):


(10) $$\begin{array}{l} Recall_{i}=\frac{h_{ii}}{\sum_{t=1}^{I}h_{it}}, \end{array}$$

(11) $$\begin{array}{l} Precision_{i}=\frac{h_{ii}}{\sum_{t=1}^{I}h_{ti}}, \end{array}$$

(12) $$\begin{array}{l} Accuracy=\frac{\sum_{t=1}^{I}h_{ii}}{N}. \end{array}$$

**Table 6:** Multi-classification confusion matrix.

	**Mode *i***	**Predictive class**					**Recall**
		**1**	**2**	**3**	**…**	***I***	
Actual class	1	*h_11_*	*h_12_*	*h_13_*	…	*h_1*I*_*	Recall_1_
	2	*h_21_*	*h_22_*	*h_23_*	…	*h_2*I*_*	Recall_2_
	3	*h_31_*	*h_32_*	*h_33_*	…	*h_3*I*_*	Recall_3_
	…	…	…	…	…	…	
	*I*	*h_*I*1_*	*h_*I*2_*	*h_*I*3_*		*h_*II*_*	Recall_S_
	Precision	Precision_1_	Precision_2_	Precision_3_		Precision_S_	Accuracy

## Results

### Determination of variables

Before data analysis, a multicollinearity test was conducted on the relevant independent variables, and no obvious collinearity relationships were revealed (Table 7).

**Table 7:** Multicollinearity test results.

**Variables**	**VIF**	**1/VIF**
Intercity travel cost	2.31	0.43365
Intercity travel distance	1.93	0.517114
Intercity mode of transportation	1.78	0.562454
Relative humidity	1.44	0.694347
Personal monthly income	1.25	0.801631
Rainfall	1.22	0.82139
Temperature	1.21	0.829228
Visibility	1.19	0.837374
Air quality index	1.17	0.851836
Age	1.13	0.881774
Occupation	1.12	0.896377
Car ownership	1.1	0.906249
Wind power	1.09	0.919628
Intercity travel time	1.07	0.932903
Travel purposes	1.05	0.949109
Gender	1.05	0.956899
Mean VIF	1.32	

### Parameter estimation and feature importance

The ordered logit model (OLM) parameter estimation results are shown in Table 8, where the coefficient reflects the influence degree of the specific explanatory variable on the dependent variable. Furthermore, a positive (negative) regression coefficient indicates that the occurrence ratio will increase (decrease) when the explanatory variable increases by one unit.

**Table 8:** Parameter estimation from OLM.

**Variables**	**Happiness degree**			**Panic degree**			**Anxiety**			**Tiredness degree**		
	**Coefficient**	***P* > *Z***	**OR**	**Coefficient**	***P* > *Z***	**OR**	**Coefficient**	***P* > *Z***	**OR**	**Coefficient**	***P* > *Z***	**OR**
**Gender**												
Male vs. female	−0.164	0.05	0.849	0.198	0.018	1.219	/	/	/	/	/	/
**Age**												
Over 60 years old vs. 30–60	−0.29	0.036	0.748	/	/	/	/	/	/	0.32	0.018	1.377
**Occupation**												
Institution personnel vs. Enterprise units	−0.579	0	0.56	−0.27	0.032	0.763	/	/	/	/	/	/
Students vs. Enterprise units	−0.211	0.03	0.809	/	/	/	/	/	/	/	/	/
Farmers vs. Enterprise units	/	/	/	−0.656	0	0.519	/	/	/	/	/	/
Self-employed vs. Enterprise units	/	/	/	−0.345	0.029	0.708	/	/	/	0.431	0.007	1.539
Other vs. Enterprise units	/	/	/	−0.321	0.019	0.726	−0.319	0.017	0.727	/	/	/
*Personal monthly income*	/	/	/	−0.051	0.036	0.95	/	/	/	/	/	/
**Car ownership**												
Yes vs. No	−0.279	0.001	0.757	0.303	0	1.354	0.37	0	1.447	0.217	0.011	1.242
**Temperature**												
≥25°C vs. 15–25°C	−0.717	0	0.488	1.286	0	3.619	0.964	0	2.621	0.089	0.649	1.093
10–15°C vs. 15–25°C	−0.343	0.001	0.71	0.58	0	1.785	0.64	0	1.896	0.814	0	2.256
5–10°C vs. 15–25°C	−0.254	0.086	0.776	0.507	0.001	1.661	0.654	0	1.924	0.947	0	2.577
≤ 0°C vs. 15–25°C	−1.462	0	0.232	1.034	0	2.813	1.086	0	2.963	1.143	0	3.136
*Rainfall*	/	/	/	0.034	0.045	1.035	0.045	0.018	1.046	0.057	0.011	1.058
*Relative humidity*	0.01	0	1.01	/	/	/	/	/	/	−0.004	0.048	0.996
*Wind power*	−0.146	0.001	0.864	0.161	0	1.174	0.201	0	1.223	0.175	0	1.191
*Air quality index*	−0.006	0	0.994	0.009	0	1.009	0.006	0	1.006	0.002	0.024	1.002
*Visibility*	0.003	0.041	1.003	−0.001	0.053	0.999	/	/	/	−0.002	0	0.998
**Travel purposes**												
Leisure travel vs. Mandatory travel	0.46	0	1.585	−0.245	0.004	0.783	−0.232	0.005	0.793	−0.214	0.01	0.808
**Intercity travel mode**												
Airplane vs. HSR	/	/	/	−0.764	0	0.466	−0.649	0	0.523	−0.438	0	0.645
Bullet train vs. HSR	−0.683	0	0.505	/	/	/	/	/	/	/	/	/
Train vs. HSR	0.635	0	1.888	−1.029	0	0.357	0.415	0	1.515	−0.309	0.008	0.734
Express bus vs. HSR	/	/	/	/	/	/	−0.768	0	0.464	−0.365	0.007	0.695
*/cut1*	−2.624			−1.210			−1.612			−0.644		
*/cut2*	−0.778			0.153			−0.144			0.879		
*/cut3*	1.400			1.531			1.214			2.340		
*/cut4*	4.257			3.866			3.573			4.549		

**Figure 1:**
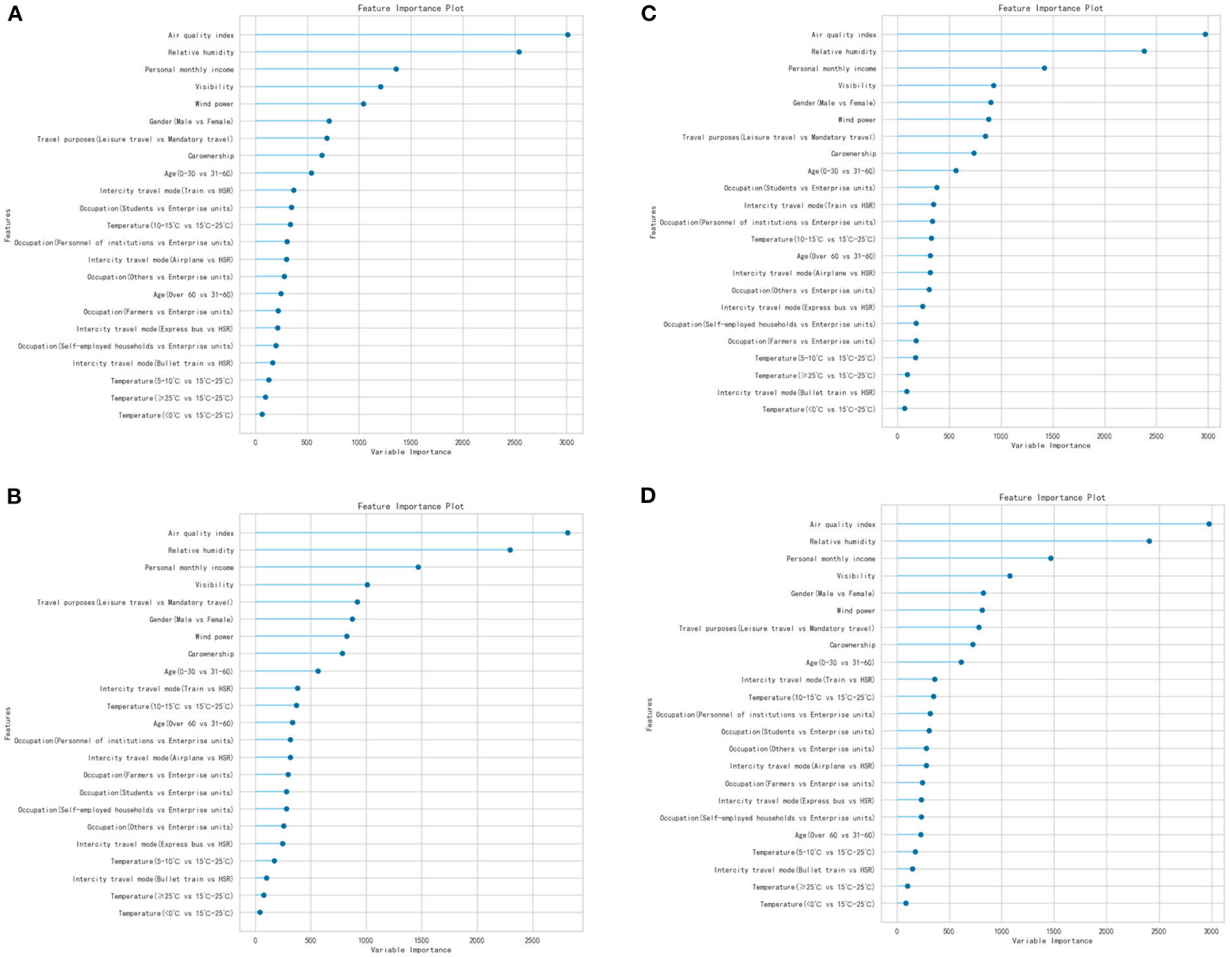
Importance analysis of support vector machine (SVM). **(A)** Happiness degree. **(B)** Panic degree, **(C)** Anxiety degree, **(D)** Tiredness degree.

In addition, the significant influencing factors in the OLM were taken as the input features, and an SVM was used to build a model for each of the four dependent variables. After the model construction and calibration, a 4-fold cross-validation and hyperparameter tuning were applied to produce the optimal training model, and the conclusions of the models evaluation are shown in Table 9; whereas the feature importance of the four SVM models is shown in Figure 1.

**Table 9:** Confusion matrix.

**Model**			**Mode**	**Calculation set**						**Validation set**					
				**Predictive class**						**Predictive class**					
				**1**	**2**	**3**	**4**	**5**	**Recall**	**1**	**2**	**3**	**4**	**5**	**Recall**
Ordered logistic model	Happiness	Actual class	1	3	85	98	0	0	1.61%	4	35	15	0	0	7.41%
			2	6	144	318	0	0	30.77%	4	76	55	1	0	55.88%
			3	2	120	561	1	0	82.02%	1	42	108	4	0	2.58%
			4	2	19	232	3	0	1.17%	0	9	53	1	0	1.59%
			5	0	6	14	0	0	0.00%	0	4	2	0	0	0.00%
			Precision	23.08%	38.50%	45.87%	75.00%	0.00%	**44.05%** (Accuracy)	44.44%	45.78%	46.35%	16.67%	0.00%	**45.65%** (Accuracy)
	Panic	Actual class	1	0	11	74	37	1	0.00%	0	7	13	4	0	0.00%
			2	0	37	89	94	3	16.59%	2	14	23	15	1	25.45%
			3	0	13	109	268	5	27.59%	0	7	43	51	4	40.95%
			4	1	7	79	556	17	84.24%	0	3	17	140	9	82.84%
			5	0	0	22	181	10	4.69%	0	0	4	52	5	8.20%
			Precision	0.00%	54.41%	29.22%	48.94%	27.78%	**44.11%** (Accuracy)	0.00%	45.16%	43.00%	53.44%	26.32%	**48.79%** (Accuracy)
	Anxiety	Actual class	1	0	9	67	38	1	0.00%	0	7	12	3	0	0.00%
			2	0	27	94	121	0	11.16%	0	21	30	11	1	33.33%
			3	0	19	121	273	1	29.23%	0	6	29	68	2	27.62%
			4	0	9	80	574	3	86.19%	0	7	24	132	3	79.52%
			5	0	1	17	158	1	0.56%	0	0	7	48	3	5.17%
			Precision	0.00%	41.54%	31.93%	49.31%	16.67%	**44.80%** (Accuracy)	0.00%	51.22%	28.43%	50.38%	33.33%	**44.69%** (Accuracy)
	Tiredness	Actual class	1	0	3	120	17	0	0.00%	0	6	16	5	0	0.00%
			2	0	21	207	76	0	6.91%	0	17	48	29	0	18.09%
			3	1	17	331	167	0	64.15%	0	17	59	48	0	47.58%
			4	0	3	231	279	0	54.39%	0	6	40	82	0	64.06%
			5	0	1	43	78	0	0.00%	0	3	11	18	0	0.00%
			Precision	0.00%	46.67%	35.52%	45.22%	0.00%	**39.56%** (Accuracy)	0.00%	34.69%	33.91%	45.05%	0.00%	**39.01%** (Accuracy)
SVM	Happiness	Actual class	1	100	43	37	6	0	53.76%	17	20	17	0	0	31.48%
			2	15	321	103	29	0	68.59%	17	64	38	17	0	47.06%
			3	11	79	546	47	1	6.87%	8	38	80	28	1	51.61%
			4	5	24	75	152	0	55.00%	0	12	20	31	0	49.21%
			5	1	1	5	2	11	55.00%	1	3	2	0	0	0.00%
			Precision	75.76%	68.59%	71.28%	64.41%	91.67%	**70.01%** (Accuracy)	39.53%	46.72%	50.96%	40.79%	0.00%	**46.38%** (Accuracy)
	Panic	Actual class	1	70	13	18	21	1	56.91%	5	5	9	4	1	20.83%
			2	20	123	29	43	8	55.16%	4	24	10	17	0	43.64%
			3	14	27	231	107	16	58.48%	7	8	27	44	19	25.71%
			4	18	16	46	557	23	84.39%	7	9	22	110	21	65.09%
			5	3	6	22	60	122	57.28%	0	3	15	24	19	31.15%
			Precision	56.00%	66.49%	66.76%	70.69%	71.76%	**68.34%** (Accuracy)	21.74%	48.98%	32.53%	55.28%	31.67%	**44.69%** (Accuracy)
	Anxiety	Actual class	1	35	5	47	27	1	30.43%	3	5	9	5	0	13.64%
			2	8	78	75	75	6	3.31%	3	22	22	14	2	34.92%
			3	12	30	173	193	6	41.79%	4	8	23	63	7	21.90%
			4	16	11	77	554	8	83.18%	4	4	26	124	8	74.70%
			5	2	6	30	90	49	27.68%	1	1	5	42	9	15.52%
			Precision	47.95%	60.00%	43.03%	59.00%	70.00%	**55.08%** (Accuracy)	20.00%	55.00%	27.06%	50.00%	34.62%	**43.72%** (Accuracy)
	Tiredness	Actual class	1	54	24	33	26	3	38.57%	3	8	8	8	0	11.11%
			2	7	151	104	39	3	49.67%	4	27	38	19	6	28.72%
			3	14	41	377	74	10	73.06%	10	18	60	29	7	48.39%
			4	16	33	99	357	8	69.59%	4	13	46	62	3	48.44%
			5	0	12	41	15	54	44.26%	2	3	17	5	5	15.63%
			Precision	59.34%	57.85%	57.65%	69.86%	69.23%	**62.26%** (Accuracy)	13.04%	39.13%	35.50%	50.41%	23.81%	**38.77%** (Accuracy)

### Model interpretation

#### Factors from individual's socioeconomic background

According to the OR values and importance of factors (Table 8; Figure 1), the impact of each significant variable on passenger mood during intercity travel was analyzed. Firstly, gender (male vs. female) was found to have a significant impact on the degrees of happiness and panic of passengers, both ranking the 6th place in importance according to the results of the SVM; whereas the OR values of happiness and panic degrees were 0.849 and 1.219, respectively, indicating that the odds of male passengers' happiness toward intercity travel would decrease 15.1% compared with females, while an additional level of passengers' panic for males would increase 21.9%. One possible reason is that males assume more social responsibilities in Chinese society, and feel greater pressure from both work and home life (60); thus, in general, they might suffer from more likelihood to be less happy than women, and even more anxious when traveling between cities.

Age (>60 vs. 30–60) was also found to have a significant impact on passenger happiness and tiredness during intercity travel according to the OLM. The SVM model showed that the importance of age ranked the 16th and 19th for happiness and tiredness, respectively. The OR value of the variable for happiness showed that the odds of an additional level of happiness for passengers over 60 would decrease by 25.2% in contrast to passengers aged 30–60 years-old; whereas, the odds of an additional level of tiredness for passengers > 60 years-old would increase by 37.7%. This is intuitive as more elderly passengers typically have less energy than their middle-aged counterparts, both physically and mentally; thus, they are more likely to feel tired during long-distance travel.

Occupation (institution personnel vs. enterprise personnel) showed a significant negative correlation with travel happiness and panic in the OLM results, with the SVM model revealing the 13th place in terms of importance for both mood factors. In addition, the OR values of occupation were 0.56 and 0.763 for institution personnel and enterprise units, respectively, suggesting that the odds of an additional degree of happiness perceived by institution personnel would reduce by 34%, and the odds of an additional degree of panic would decrease by 23.7%. This might be related to the travel system of the public institutions maintaining strict regulations, with limited choices of travel mode; thus, these employees would likely experience a corresponding reduction in happiness during travel.

Similarly, occupation (students vs. enterprise personnel) had a significantly negative effect on passengers' intercity travel happiness, while that of farmers vs. enterprise units and self-employed vs. enterprise units retained a significantly negative effect on passenger's panic feelings. Here, the OR value for occupation (students vs. enterprise units) was 0.809, suggesting that the odds of an additional degree of happiness perceived by the students would reduce by 19.1%; whereas the OR values for panic were 0.519 and 0.708, respectively, indicating that the odds of an additional degree of passenger panic toward intercity travel for farmers would decrease by 48.1% compared with enterprise units, while that for self-employed passengers would decrease by 29.2%. This could be a result that hinges on the very limited choices for students due to their financial constraints. For example, for those college and university students from a low-income household, they are more willing to trade a lower travel cost with sacrifice of comfortness in long-distance travel (this is quite typical in China), thus, leading to a relatively lower degree of happiness during travel.

Personal monthly income also had a negative correlation with traveler panic according to the ordered logistic regression, with the SVM model indicating it ranked the 3rd in importance. Further, the OR value of 0.95 indicated that the odds of an additional degree of passenger panic would decrease by 5% for every 1,000¥ increase in personal monthly income. This may derive from passengers with higher incomes often choosing safer and more efficient modes of travel (e.g., airplanes and HSRs), reducing their panic degree during intercity travel.

Car ownership was also identified to have a significant impact on the happiness, panic, anxiety, and tiredness of passengers during intercity travel according to the regression results. The OR values of car ownership for these four factors were 0.757, 1.354, 1.447, and 1.242, respectively, indicating that the odds of an additional degree of happiness for passengers with cars would decrease by 24.3% compared to those without cars, while additional degrees of panic, anxiety and tiredness would increase by 35.4, 44.7, and 24.2%, respectively. This might be related to the fact that passengers with cars often go to airports or stations by driving their own private cars, and thus, facing the additional mental pressures of finding parking lots and paying for a longer parking period, which might lead to an increasing level of anxiety, panic, and exhaustion.

#### Factors relate to weather conditions

The external temperature was the most critical factor influencing passenger mood during intercity travel, ranking the 12th, 11th, 13th, and 11th in the importance for happiness, panic, anxiety, and tiredness, respectively. The OR value for temperatures: ≥25°C vs. 15–25°C, 10–15°C vs. 15–25°C, 5–10°C vs. 15–25°C), and ≤ 0°C vs. 15–25°C) were 0.488, 0.71, 0.776, 0.232, respectively, showing that compared with the passengers traveling in temperature of 15–25°C, the odds of additional degrees of happiness perceived by passengers traveling at temperatures 25°C, 10–15°C, 5–10°C, and ≤ 0°C would decrease by 51.2, 29.0, 22.4, and 76.8%, respectively. In addition, temperature showed a significant effect on passenger panic, and the OR values of this indicated that the odds of an additional degree of panic perceived by passengers traveling in temperatures >25°C, 10–15°C, 5–10°C, and ≤ 0°C would increase by 261.9, 78.5, 66.1, and 181.3%, respectively. Similarly, the odds of an additional degree of anxiety perceived by passengers traveling in temperatures >25°C, 10–15°C, 5–10°C, and ≤ 0°C would increase by 162.1, 89.6, 92.4, and 196.3%, respectively.

Moreover, the odds of additional degrees of tiredness being perceived by passengers traveling in temperatures >25°C, 10–15°C, 5–10°C, and ≤ 0°C would increase by 9.3, 125.6, 157.7, and 213.6%, respectively. This indicates that the external temperatures between 15 and 25°C showed a significantly positive effect on passenger emotion, which may be derived from the additional comfort provided by environments between 15 and 25°C.

Rainfall is another generally important characteristic variable impacting travel mood, resulting in a series of OR values of 1.035, 1.046, and 1.058, indicating that the odds of additional degrees of panic, anxiety, and tiredness would increase by 3.5, 4.6, and 5.8%, respectively. This is likely because rainfall would directly affect the operation of intercity traffic modes, as well as passengers' moods. Specifically, an increase in rainfall could likely lead to a large-scale delays, while extreme rainfall may even lead to disruptions and cancellations. This will inevitably increase passengers' anxiety, mental tension, and exhaustion to a higher extent. Such findings are supported by Wu and Liao (26), who showed that extreme rainfall can significantly impact passengers' travel moods and reduce travel demand.

Relative humidity also displayed significant positive and negative impacts on happiness and tiredness, respectively, with corresponding OR values of 1.01 and 0.996, indicating that the odds of the additional degrees of happiness would increase by 1.0%, while that of additional tiredness would decrease by 0.4%. The ranking results of the importance degree, however, showed that relative humidity had little influence on passengers' happiness and tiredness degrees in intercity travel.

Furthermore, wind speed ranks the 5th, 7th, 6th, and 6th, respectively, while the OR values of wind speed for happiness, panic, anxiety, and tiredness are 0.864, 1.174, 1.223, and 1.191 respectively, indicating that the odds of an additional degree of passengers' happiness would be decreased by 13.6% with every unit of increment in wind speed. In addition, the increase of one unit of wind speed will increase the odds of the additional level of passengers' panic, anxiety, and tiredness degree by 17.4, 22.3, and 19.1%, respectively. Intuitively, a windy weather would lead to higher anxiety, panic, and even fatigue in long-distance travel, thereby reducing their happiness. This can be supported by the findings from Yazdanpanah and Hosseinlon (24) and Alberto et al. (25). Similarly, the air quality was also found to be an important variable impacting passengers' emotions during intercity travel. The OR value for happiness on this factor was 0.994, showing that each additional unit of the air quality index decreased the odds of additional degrees of happiness by 0.6%. The odds of additional degrees of panic, anxiety, and tiredness increased by 9, 6, and 2%, respectively. The higher the air quality index, the worse the air quality is, which is very intuitive to realize its effects on the travel mood.

Visibility was similarly revealed to have a measurable impact on intercity passenger happiness, panic, and tiredness, all ranking the 4th according to the SVM models. An OR value for happiness degree of 0.994 showed that an increase of each additional unit of visibility increased the odds of additional degrees of happiness by 3%. Additionally, the odds level of passengers' panic and tiredness decreased by 1 and 2%, respectively. In general, what we can see from the above is that a good weather condition, with clean air, moderate temperature and wind, and clear vision, would significantly produce good emotional feelings in long-distance travel.

#### Factors of travel characteristics

Travel purpose was similarly discovered to have a significant effect on happiness, panic, anxiety, and tiredness, ranking the 7th, 5th, 7th, and 7th, respectively. Specifically, the OR values of travel purpose were 1.585, 0.783, 0.793, and 0.808, respectively, indicating that compared with mandatory travel, the odds of the additional degrees of happiness perceived by passengers engaging in leisure travel can increase by 58.5%, while those of for panic, anxiety, and tiredness would decrease by 21.7, 20.7, and 19.2%, respectively. This also seems relatively intuitive, as passengers involved in leisure travel are more prone to feel happy, and less likely to suffer from panic, anxiety, and tiredness.

Lastly, intercity mode of transportation (airplane vs. HSR) was an essential characteristic variable in travel mood, ranking the 10th, 10th, 11th, and 10th in happiness, panic, anxiety, and tiredness according to the SVM. The OR values for intercity travel mode (airplane vs. HSR) were 0.466, 0.523, and 0.645, respectively, indicating that compared with HSR, the odds of an additional degree of panic, anxiety, and tiredness would decrease by 53.4, 47.7, and 35.5%. This is likely related to the greater travel speed, and efficiency of air travel, as has been shown by Masson and Petiot (27), where under the same time conditions, passengers tended to choose the mode of transportation with a faster speed to reduce travel time.

Alternatively, the OR values of intercity transportation mode (train vs. HSR) were 1.888, 0.357, 1.515, and 0.734, respectively, indicating that compared with HSR, the odds of additional degrees of happiness and anxiety perceived by passengers would increase by 88.8 and 51.5%; whereas those of panic and tiredness would decrease by 64.3 and 26.6%, respectively. In particular, we found that HSR was associated with negative emotions compared with other modes of transportation. One possible reason could lie in the poor inter-connections among various functional areas at the HSR stations in most of the major Chinese cities (such as arrival area, ticketing area, security check points, transfer area, waiting zones, and other basic facilities). China's major cities have now all equipped with new HSR stations that built with overhead suspension truss-like structures with massive open space and long overarching internal span distance, and various functional areas are often arranged on different floors, which leads to long walking time for passengers. This can easily lead to negative emotions among passengers traveling by HSRs, especially those with heavy carried luggages. Interestingly, we found that this finding is actually inconsistent with St-Louis et al. (29), who found that the positive mood of pedestrians, train commuters, and cyclists was significantly higher than that of drivers or subway and bus users, which could be due to the significant differences between HSR and traditional trains in China.

## Discussion

From the results, several practical implications for the development of intercity transportation and environmental protection can be inferred. First of all, the result shows that weather have a crucial impact on the mood of passengers during inter-city travel. A lower PM2.5 concentration (i.e., a better air quality) and wind speed, suitable temperature (20–25°C) and higher visibility are closely related to the positive travel mood of passengers. Given the current circumstances in China, it suggests that the protection of the urban environment is still an important work in the process of China's rapid urbanization and strategic development, which aims to effectively improve the social wellbeing and health of the citizens in a long run.

In addition, we found that the travel mode can also affect the travel mood of the passengers. Surprisingly, passengers traveling by HSR are more likely to be associated with negative travel moods than those passengers traveling by other transportation modes. One of the implications could be that the design of the functional divisions at HSR stations in China should be further improved with better inter-connections and smoother internal transitions. This deficiency might lead to some negative experience, such as a long walking distance for passengers, especially with heavy luggages during inter-city trips. This shows, to a large extent, that the efficient inter-connection of various functional areas of high-speed railway stations, such as the entrance and exit of HSR stations, ticket counters, waiting areas, car-parking facilities and taxi parking areas, could be a managerial focus for station management in the future.

The findings on the car ownership reveals that the travel emotion of passengers with cars is surprisingly lower in intercity travel. Given the possible reason that this might be due to the difficulties and burdens from parking issues at the stations, optimizing the design of parking lots seems to be a fairly reasonable measure. By providing more parking spaces connecting with intercity transportation hubs, it could be an effective and efficient measure to improve the travel emotion of those particular passengers. For example, a better sign design in the parking lobby and signal indicators of available parking lots could be considered here for this particular issue, and we have witnessed that suchlike design has been widely implemented at many transportation hubs in recent years in China. Finally, given the prominent phenomenon of rapid aging populations in China, the elderly over 60 are more likely to suffer from unhappiness and tiredness, which suggests that developing more effective measures to provide extra care for the elderly travelers in the intercity long-distance mobility could be a good managerial policy and should be considered by the local authorities, the government, and transportation service providers.

## Conclusions

The present study analyzes the factors affecting passengers' moods in intercity travel in Xi'an city, including individual socioeconomic characteristics, transportation modes, weather conditions, and passengers' moods. Four dimensions—happiness, panic, anxiety, and tiredness—are divided into five levels to represent an objective evaluation system for assessing passenger mood conditions during intercity travel. Using the OLM and SVM models in tandem to establish the regression relationship between the four different dimensions of passenger moods and independent variables, the major determinants, and importance of these influential factors are explored and several policy and managerial implications are put forward. The main conclusions of this study can be summarized as follows.

(1) By applying the ordered logit regression model to analyze and identify the influential factors affecting passengers' moods during intercity travel, it was revealed that gender, age, occupation, personal monthly income, car ownership, temperature, rainfall, relative humidity, wind speed, air quality index, visibility, travel purpose, intercity mode of transportation, and intercity travel time all have significant effects on the different dimensions of passengers' moods, including happiness, panic, anxiety, and tiredness.
(2) Compared with females, males were more prone to feel panic, and less likely to feel happiness during intercity travel. Compared to passengers of age 30–60, individuals with an age of over 60 were less happy when traveling and more likely to experience tiredness. Compared with enterprise units, institution personnel and students maintained a lower sense of happiness during travel, while self-employed households were more prone to tiredness. Furthermore, for every ¥1,000, increase in monthly income, the probability of passenger panic can be reduced by 5%. For weather conditions, temperatures between 15 and 25°C, lower wind speeds, greater relative humidity, lower air quality index (i.e., better air quality), higher visibility, and lower precipitation all bettered the moods of intercity passengers (i.e., increasing their sense of happiness). Compared to mandatory travel, passengers engaged in leisure travel were more likely to feel happy, and less likely to feel panic, anxiety, and tiredness.
(3) To explore the different effects of independent variables on passengers' moods during intercity travel, SVMs were used to calculate the contribution of independent variables (i.e., the relative importance of features), where the higher the relative importance value, the greater the contribution to estimating the dependent variable. These results showed that the most important variable affecting happiness was occupation (23% importance); whereas those for panic and anxiety were intercity transportation mode (38 and 39%, respectively), while the most important variable affecting tiredness was the weather temperature (22%).

Finally, we also acknowledge three limitations of this study: (1) only one city in China was investigated as the case study, yet factors affecting passengers' emotional perceptions and wellbeing could be affected by different cultural backgrounds and urban environments. Hence, additional data from other cities with various geographical contexts should be further investigated in the future to verify the sensitivity of the findings. (2) Even though it would not alter the main findings and conclusions, it should still be noted that the survey data on multi-classes could be slightly imbalanced due to random sampling, therefore, the prediction accuracy of models was only at moderate and acceptable levels. Statistically, this issue can be addressed in future studies by adopting over- or under-sampling methods to re-balance the classes. (3) It is also noteworthy that passengers traveling to one city as the destination and those passengers leaving this city as the origin might have slightly different moods when carrying out their long-distance intercity travels, which might cause some potential estimation bias. Likewise, other unpredictable coincidences and various trivial, yet dynamic, influencing factors, such as instantaneous perceptions, could also affect the overall travel moods, which can hardly be observed through questionnaire surveys. The future work could explore more on these issues and interesting findings might be further revealed.

## Data availability statement

The datasets presented in this article are not readily available because the authors do not have permission to share the data. However, metadata might be available to share upon request. Requests to access the datasets should be directed to lixiaowei@xauat.edu.cn.

## Author contributions

Conceptualization, methodology, data curation, validation, and software: XL, YW, and JT. Writing original draft, investigation, and writing review and editing: XL, YW, JT, LS, and TZ. Funding acquisition and supervision: XL, JT, and JC. All authors contributed to the article and approved the submitted version.

## References

[1] Ministry of Transport of China (2020). Statistical Bulletin on the Development of China's Transport Industry. Beijing: Ministry of Transport of China (2021).

[2] Bown J White C. Affect in a self-regulatory framework for language learning. System. (2010) 38:432–43. 10.1016/j.system.2010.03.016

[3] Bigné J Andreu L. Emotions in segmentation: an empirical study. Ann Tour Res. (2004) 31:682–96. 10.1016/j.annals.2003.12.01836293893

[4] Bel L Jordan L. Beyond the friendly skies: an integrative framework for managing the air travel experience. J Serv Theory Pract. (2005) 15:437–51. 10.1108/09604520510617293

[5] Jonas L. Tourism mobilities and the travel glance: experiences of being on the move. Scand J Hosp Tour. (2001) 1:80–98. 10.1080/150222501317244010

[6] Stradling S Carreno M Rye T Noble A. Passenger perceptions and the ideal urban bus journey experience. Transp Policy. (2007) 14:283–92. 10.1016/j.tranpol.2007.02.003

[7] Rui C Patricio L Jorge N Magee C. Understanding the travel experience and its impact on attitudes, emotions and loyalty towards the transportation provider a quantitative study with mid-distance bus trips. Transp Policy. (2014) 31:35–46. 10.1016/j.tranpol.2013.11.006

[8] Rajesh I Daruri V. The impact of social cues on passengers' travel experience. Serv Indust J. (2019) 39:299–318. 10.1080/02642069.2018.1521389

[9] Meenar M Flamm B Keenan K. Mapping the emotional experience of travel to understand cycle-transit user behavior. Sustainability. (2019) 11:4743. 10.3390/su11174743

[10] Bocker L Dijst M Faber J. Weather, transport mode choices and emotional travel experiences. Transp Res A. (2016) 94:360–73. 10.1016/j.tra.2016.09.021

[11] Beam C Mingus C Watkins K. An adaption of the level of traffic stress based on evidence from the literature and widely available data. Res Transp Bus Manag. (2018) 29:50–62. 10.1016/j.rtbm.2018.12.002

[12] Corvec S Zhao J. Transport and emotion: how neurosciences could open a new research field. Travel Behav Soc. (2020) 20:12–21. 10.1016/j.tbs.2020.02.001

[13] Schneider C Zaval L Markowitz E. Positive emotions and climate change. Curr Opin Behav Sci. (2021) 42:114–20. 10.1016/j.cobeha.2021.04.009

[14] Jodas S Marranghello N Pereira S Guido C. Comparing support vector machines and artificial neural networks in the recognition of steering angle for driving of mobile robots through paths in plantations. Procedia Comput Sci. (2013) 18:240–9. 10.1016/j.procs.2013.05.187

[15] Bedi P Mewada S Vatti A Singh C Sikarwar R. Detection of attacks in IoT sensors networks using machine learning algorithm. Microproc Microsyst. (2021) 82:103814. 10.1016/j.micpro.2020.103814

[16] Saidi L Ali B Fnaiech F. Application of higher order spectral features and support vector machines for bearing faults classification. ISA Trans. (2015) 54:193–206. 10.1016/j.isatra.2014.08.007 25282095

[17] Elangovan M Sugumaran V Ramachandran I Ravikumar S. Effect of SVM kernel functions on classification of vibration signals of a single point cutting tool. Expert Syst Appl. (2011) 38:15202–7. 10.1016/j.eswa.2011.05.081

[18] Salem B Bacha K Chaari A. Support vector machine based decision for mechanical fault condition monitoring in induction motor using an advanced hilbert-park transform. ISA Trans. (2012) 51:566–72. 10.1016/j.isatra.2012.06.002 22742760

[19] Liu Z Wei G Hu J Ma W. A hybrid intelligent multi-fault detection method for rotating machinery based on RSGWPT, KPCA and twin SVM. ISA Transactions. (2017) 66:249. 10.1016/j.isatra.2016.11.001 27837907

[20] Sugumaran V Muralidharan V Ramachandran KI. Feature selection using decision tree and classification through proximal support vector machine for fault diagnostics of roller bearing. Mech Syst Signal Process. (2007) 21:930–42. 10.1016/j.ymssp.2006.05.004

[21] Palma K. The impact of adverse weather conditions on the propensity to change travel decisions: a survey of Brussels commuters. Transp Res Part A Policy Pract. (1997) 31:181–203.

[22] Delaplace M Dobruszkes F. From low-cost airlines to low-cost high-speed rail? The french case. Transport Policy. (2015) 38:73–85. 10.1016/j.tranpol.2014.12.006

[23] Morris A Guerra E. Mood and mode: does how we travel affect how we feel? Transportation. (2015) 42:25–43. 10.1007/s11116-014-9521-x

[24] Yazdanpanah M Hosseinlou MH. The influence of personality traits on airport public transport access mode choice: a hybrid latent class choice modeling approach. J Air Transp Manag. (2016) 55:147–63. 10.1016/j.jairtraman.2016.04.010

[25] Alberto M Tim JR. The impact of extreme weather conditions on long distance travel behaviour. Transp Res A Policy Pract. (2015) 77:305–19. 10.1016/j.tra.2015.04.025

[26] Wu J Liao H. Weather, travel mode choice, and impacts on subway ridership in Beijing. Transp Res A Policy Pract. (2020) 135:264–79. 10.1016/j.tra.2020.03.020

[27] Masson S Petiot R. Can the high speed rail reinforce tourism attractiveness? The case of the high speed rail between Perpignan and Barcelona. Technovation. (2009) 29:611–7. 10.1016/j.technovation.2009.05.013

[28] Harvey J Thorpe N Caygill M Namdeo A. Public attitudes to and perceptions of high speed rail in the UK. Transport Policy. (2014) 36:70–8. 10.1016/j.tranpol.2014.07.008

[29] St-Louis E Manaugh K Lierop DV El-Geneidy A. The happy commuter: a comparison of commuter satisfaction across modes. Transp Res F Psychol Behav. (2014) 26:160–70. 10.1016/j.trf.2014.07.004

[30] Bezerra G Gomes CF. The effects of service quality dimensions and passenger characteristics on passenger's overall satisfaction with an airport. J Air Transp Manag. (2015) 44–45, 77–81. 10.1016/j.jairtraman.2015.03.001

[31] Bellizzi G Eboli L Forciniti C Mazzulla G. Air transport passengers' satisfaction: an ordered logit model. Transp Res Procedia. (2018) 33:147–54. 10.1016/j.trpro.2018.10.087

[32] Bogicevic V Bujisic M Bilgihan A Yang W Cobanoglu C. The impact of traveler-focused airport technology on traveler satisfaction. Technol Forecast Soc Change. (2017) 123:351–61. 10.1016/j.techfore.2017.03.038

[33] Lu XJ Ma CX Qiao YH. Short-term demand forecasting for online car-hailing using ConvLSTM networks. Physica A Statist Mechan Appl. (2021) 570:1–11. 10.1016/j.physa.2021.125838

[34] Yang H Huo J Pan R Xie K Zhang WJ Luo X. Exploring built environment factors that influence the market share of ridesourcing service. Appl Geograp. (2022) 142:1–10. 10.1016/j.apgeog.2022.102699

[35] Ma C ZhaoY Dai G Xu X Wong SC. A novel STFSA-CNN-GRU hybrid model for short-term traffic speed prediction. IEEE Trans Intell Transp Syst. (2022) 1–10. 10.1109/TITS.2021.3117835

[36] Ma C Dai G Zhou J. Short-term traffic flow prediction for urban road sections based on time series analysis and LSTM_BILSTM method. IEEE Trans Intellig Transp Syst. (2022) 23:5615–24. 10.1109/TITS.2021.3055258

[37] Yang H Zhai G Yang L Xie K. How does the suspension of ride-sourcing affect the transportation system and environment? Transp Res D Transp Environ. (2022) 102:1–15. 10.1016/j.trd.2021.103131

[38] Zeng C Ma C Wang K Cui Z. Predicting vacant parking space availability: a DWT-Bi-LSTM model. Phys A Statist Mech Applicat. (2022) 599:127498. 10.1016/j.physa.2022.127498

[39] Strack R Kecman V Strack B Li Q. Sphere support vector machines for large classification tasks. Neurocomputing. (2013) 101:59–67. 10.1016/j.neucom.2012.07.025

[40] Duan Y Zou B Xu J Chen F Tang Y. OAA-SVM-MS: a fast and efficient multi-class classification algorithm. Neurocomputing. (2021) 454:448–60. 10.1016/j.neucom.2021.04.115

[41] Li X Lord D Zhang Y Xie Y. Predicting motor vehicle crashes using support vector machine models. Accid Anal Prevent. (2008) 40:1611–8. 10.1016/j.aap.2008.04.010 18606297

[42] Zhang Y Xie Y. Travel mode choice modeling with support vector machines. Transp Res Rec. (2008) 2076:141–50.33591977

[43] Allahviranloo M Recker W. Daily activity pattern recognition by using support vector machines with multiple classes. Transp Res B Methodol. (2013) 58:16–43. 10.1016/j.trb.2013.09.008

[44] Wang L Sun S Zhang K. A fast approximate algorithm for training L1-SVMs in primal space. Neurocomputing. (2007) 70:7–9. 10.1016/j.neucom.2006.11.003

[45] Yao Y Marcialis L Pontil M Frasconi P Roli F. Combining flat and structured representations for fingerprint classification with recursive neural networks and support vector machines. Pattern Recogn. (2003) 36:397–406. 10.1016/S0031-3203(02)00039-0

[46] Yang L Yu X Huang JX An A. Combining integrated sampling with SVM ensembles for learning from imbalanced datasets. Inform Proc Manag. (2011) 47:617–31. 10.1016/j.ipm.2010.11.007

[47] Li X Ma Q WangW Wang B. Influence of weather conditions on the intercity travel mode choice: a case of Xi'an. Comput Intellig Neurosci. (2021) 2021:9969322. 10.1155/2021/9969322 34475950 PMC8407973

[48] Li X Fan J Wu Y Chen J Deng X. Exploring influencing factors of passenger satisfaction toward bus transit in small-medium city in China. Discrete Dyn Nat Soc. (2020) 2020:1–11. 10.1155/2020/8872115

[49] Li X Wang W Xu C Li Z Wang B. Multi-objective optimization of urban bus network using cumulative prospect theory. J Syst Sci Compl. (2015) 28:661–78. 10.1007/s11424-015-2049-0

[50] Li X Tang J Hu X Wang W. Assessing intercity multimodal choice behavior in a touristy city: a factor analysis. J Transp Geogr. (2020) 86:102776. 10.1016/j.jtrangeo.2020.102776

[51] Vapnik N. The Nature of Statistical Learning Theory. New York, NY: Springer Verlag (1995).

[52] Abdollahi S Pourghasemi R Ghanbarian A Safaeian R. Prioritization of effective factors in the occurrence of land subsidence and its susceptibility mapping using an SVM model and their different kernel functions. Bull Eng Geol Environ. (2019) 78:4017–34. 10.1007/s10064-018-1403-6

[53] Liu Y Chen HY Zhang LM Wu XG Wang XJ. Energy consumption prediction and diagnosis of public buildings based on support vector machine learning: a case study in China. J Clean Prod. (2020) 272:1–15. 10.1016/j.jclepro.2020.122542

[54] Zhou Y Chang J Chang C Kao F Wang S Kang C. Multi-output support vector machine for regional multi-step-ahead pm2.5 forecasting. Sci Total Environ. (2018) 651:230–40. 10.1016/j.scitotenv.2018.09.111 30243160

[55] Hu L Cui J. Digital image recognition based on fractional-order-PCA-SVM coupling algorithm. Measurement. (2019) 145:150–9. 10.1016/j.measurement.2019.02.006

[56] Ding XJ Liu J Yang F Cao J. Random radial basis function kernel-based support vector machine. J Franklin Inst. (2021) 358:10121–40. 10.1016/j.jfranklin.2021.10.005

[57] Lundberg SM Lee SI. A unified approach to interpreting model predictions. Adv Neural Inform Proc Syst. (2017) 30:4765–74. 10.48550/arXiv.1705.07874

[58] Lundberg SM Erion G Chen H DeGrave A Prutkin JM Nair B. From local explanations to global understanding with explainable AI for trees. Nat Mach Intellig. (2020) 2:56–67. 10.1038/s42256-019-0138-9 32607472 PMC7326367

[59] Stehman V. Estimating standard errors of accuracy assessment statistics under cluster sampling. Remote Sens Environ. (1997) 60:258–69. 10.1016/S0034-4257(96)00176-9

[60] Muttakin MB Chatterjee B Khan A Mihret DG Roy R Yaftian A. Corporate political donations, board gender diversity, and corporate social responsibility: evidence from Australia. J Bus Res. (2022) 152:290–9. 10.1016/j.jbusres.2022.07.062

